# Liposome-Mediated Transdermal Delivery of Neferine for Enhanced Protection Against Skin Photoaging

**DOI:** 10.3390/ijms27209321

**Published:** 2026-10-20

**Authors:** Yi-Yin Kou, Wen-Jun Tang, Xin-Feng Hu, Zhuo-Ran Li, Yu-Fan Xue, Chi Zhang, Jia Zhao, Yi-Chen Zhou, Feng-Cheng Yang, Li-Wen Li, Zhi-Wei Yang, Zhuan-Li Bai

**Affiliations:** 1 Department of Plastic and Aesthetic Maxillofacial Surgery, The First Affiliated Hospital of Xi’an Jiaotong University, 277 West Yanta Road, Xi’an 710061, China; kyy2001@stu.xjtu.edu.cn (Y.-Y.K.); twj77466@163.com (W.-J.T.); wangweipang@stu.xjtu.edu.cn (X.-F.H.); zhuoranli0302@gmail.com (Z.-R.L.); q1q708@163.com (Y.-F.X.); 18292587388@163.com (C.Z.); 18652879623@163.com (J.Z.); yichen.zhou@stu.xjtu.edu.cn (Y.-C.Z.); yangfengcheng037@126.com (F.-C.Y.); 2 Key Laboratory of Resource Biology and Biotechnology in Western China, Ministry of Education, Northwest University, Xi’an 710069, China; liven@nwu.edu.cn; 3 MOE Key Laboratory for Nonequilibrium Synthesis and Modulation of Condensed Matter, School of Physics, Xi’an Jiaotong University, Xi’an 710049, China

**Keywords:** photoaging, neferine, liposomes, oxidative stress, cellular senescence, transdermal delivery

## Abstract

Chronic ultraviolet (UV) irradiation-induced skin photoaging is driven by oxidative stress, extracellular matrix degradation, and cellular senescence. To overcome the poor skin permeability and rapid clearance of neferine (Nef), PEGylated liposomes were prepared via thin-film hydration. The Nef-loaded liposomes (Lipo) were spherical, with a mean diameter of 137.90 ± 0.56 nm, a PDI of 0.117 ± 0.016, an encapsulation efficiency of 68.79% ± 1.01%, and a drug loading of 13.38% ± 0.22%. Ex vivo Franz diffusion cell assays demonstrated significantly higher cumulative transdermal permeation over 24 h for Lipo compared with free Nef, along with sustained-release behavior. In a chronic UVA-induced mouse photoaging model, epidermal thickness, dermal collagen networks, and fibroblast mitochondrial ultrastructure were restored by topical Lipo application, with superior effects over free Nef. In UVA-irradiated human dermal fibroblasts, senescence-associated β-galactosidase activity was reduced, intracellular ROS accumulation was eliminated, and SOD activity was restored by the treatment of Lipo. These redox modulations were associated with downregulation of the p53/p21 senescence signaling pathway. Collectively, the antioxidant and anti-senescence efficacy of Nef was enhanced by this liposomal nanoencapsulation strategy through improved transdermal kinetics and cellular responses, providing a functionalized nanoplatform for localized anti-photoaging therapy.

## 1. Introduction

Chronic ultraviolet (UV) radiation is the primary extrinsic driver of skin photoaging, characterized by collagen/elastin degradation, epidermal atrophy, and barrier dysfunction, which collectively accelerate intrinsic senescence and increase malignancy risk [1,2,3]. Mechanistically, photoaging involves sustained oxidative stress, DNA damage, and chronic inflammation [4,5]. Therapeutic interventions have expanded from conventional photoprotection to senolytic/senomorphic strategies targeting senescence-associated secretory phenotypes and key signaling cascades such as NF-κB and cGAS-STING [6]. In this landscape, natural plant-derived antioxidants, including green tea polyphenols, resveratrol, and proanthocyanidins, have gained attention as safe anti-photoaging candidates [7].

Neferine (Nef), a bisbenzylisoquinoline alkaloid from lotus seed embryos, exhibits broad pharmacological activities, including anti-psoriatic, neuroprotective and hepatoprotective effects [8,9,10,11]. Our prior studies demonstrated that Nef scavenges free radicals, enhances antioxidant enzyme activities, and inhibits collagen degradation in UV-exposed skin cells, positioning it as a promising dual-action (antioxidant and anti-senescence) agent for photoaging [12,13,14]. However, its unfavorable physicochemical properties, including poor aqueous solubility and relatively large molecular size, may limit stratum corneum penetration and reduce topical bioavailability, while achieving sustained retention at photoaged sites remains challenging [15,16,17].

Drug delivery systems can overcome the biopharmaceutical drawbacks of botanical agents. Cutaneous routes include topical and transdermal delivery, with the latter being explored for enhanced penetration and bioavailability [18]. Transdermal strategies are active (microneedles, electroporation, ultrasound) or passive (chemical enhancers and carriers) [19]. While chemical enhancers disrupt stratum corneum integrity [20] and physical methods require specialized equipment, vesicular carriers offer balanced permeation, controlled release, and biocompatibility [21]. Liposomes are particularly attractive due to their structural versatility, biodegradability, and low immunogenicity [22]. Lipid-based nanocarriers, owing to their lipid similarity to the epidermal barrier, facilitate skin transport [23,24,25]. Specifically, lecithin/cholesterol-based liposomes fuse with stratum corneum lipids, reducing barrier resistance [26]; hyaluronic acid-modified liposomes improve dermal delivery and anti-photoaging activity by promoting cellular uptake and suppressing MMP-1 upregulation [27].

**Figure 1:**
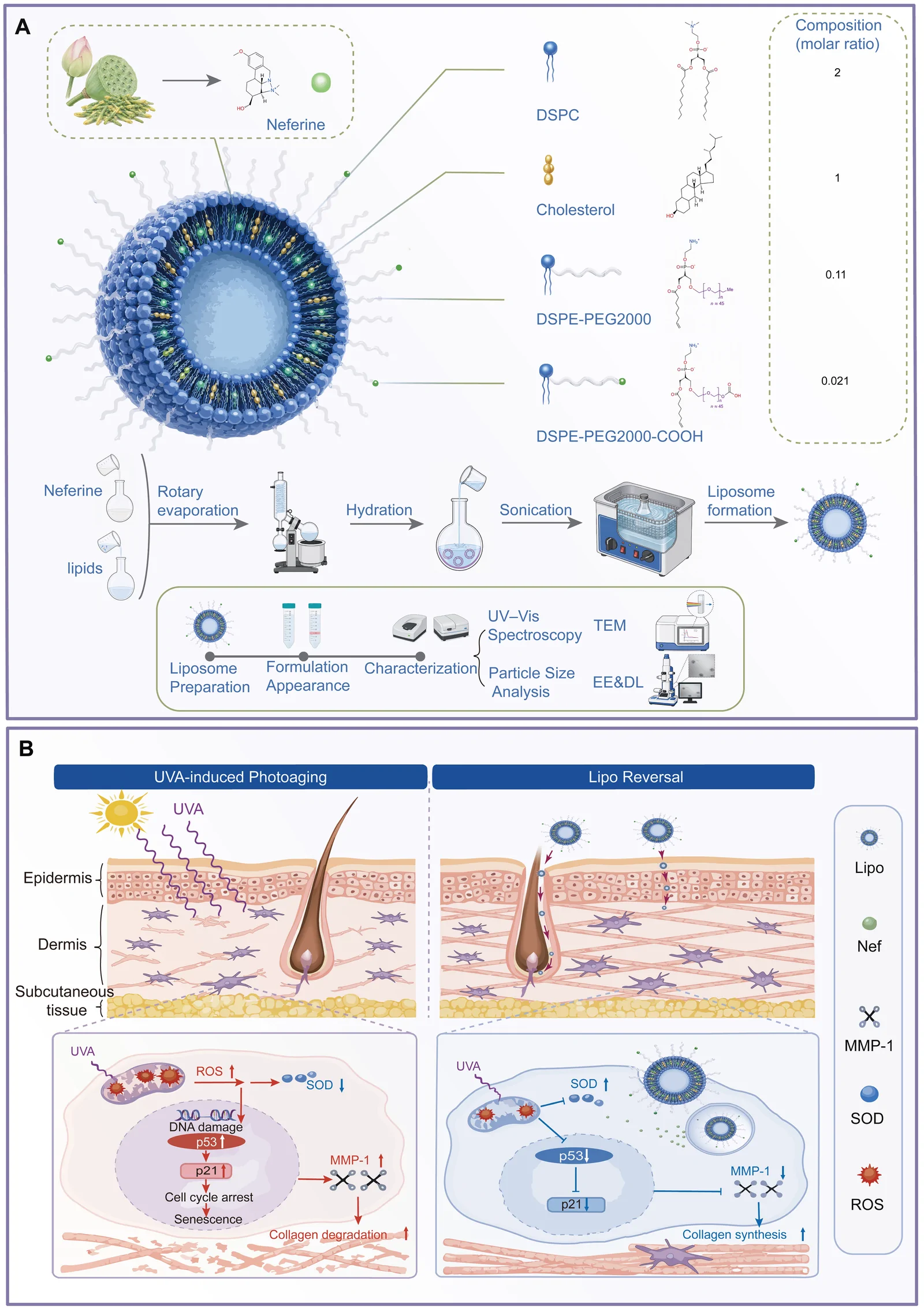
Schematic illustration of the liposomal delivery system for anti-photoaging therapy. (**A**) Composition of the Nef-loaded liposomes (Lipo), highlighting the lipid components and the encapsulation of neferine (Nef) within the lipid bilayer. Schematic diagram depicting the preparation process and the characterization methods employed for Lipo. (**B**) Proposed mechanism of transdermal delivery and anti-photoaging action. Upon topical application, Lipo penetrates the stratum corneum via the follicular route, forming a drug depot. Liposomes then release Nef, which enters dermal fibroblasts. Subsequently, Nef scavenges ultraviolet A (UVA)-induced reactive oxygen species (ROS), restores superoxide dismutase (SOD) activity, and downregulates the p53/p21 signaling pathway, thereby inhibiting cellular senescence and protecting the dermal collagen network from degradation. The figure was created by the authors using Adobe Illustrator software (version 29.1).

In this study, we constructed a PEGylated liposome encapsulating Nef via thin-film hydration (TFH), using a DSPC/cholesterol/DSPE-PEG2000/DSPE-PEG2000-COOH formulation. Nef-loaded liposomes (Lipo) (~138 nm, polydispersity index (PDI) < 0.12, encapsulation efficiency (EE) ~69%) exhibited sustained transdermal permeation in Franz cells, outperforming free Nef over 24 h. In a chronic UVA-irradiated mouse model, topical Lipo restored epidermal thickness, dermal collagen, and mitochondrial integrity, while in UVA-challenged fibroblasts, it suppressed reactive oxygen species (ROS) overproduction, restored superoxide dismutase (SOD) activity, and downregulated p53/p21 signaling. Collectively, the fabrication strategy and proposed biological effects of Lipo are illustrated in Figure 1. This liposomal platform overcomes the transdermal barrier of Nef and amplifies its antioxidant/anti-senescence efficacy, offering a promising nanotherapeutic for localized anti-photoaging intervention.

## 2. Results

### 2.1. Characterization of Liposomes

**Figure 2:**
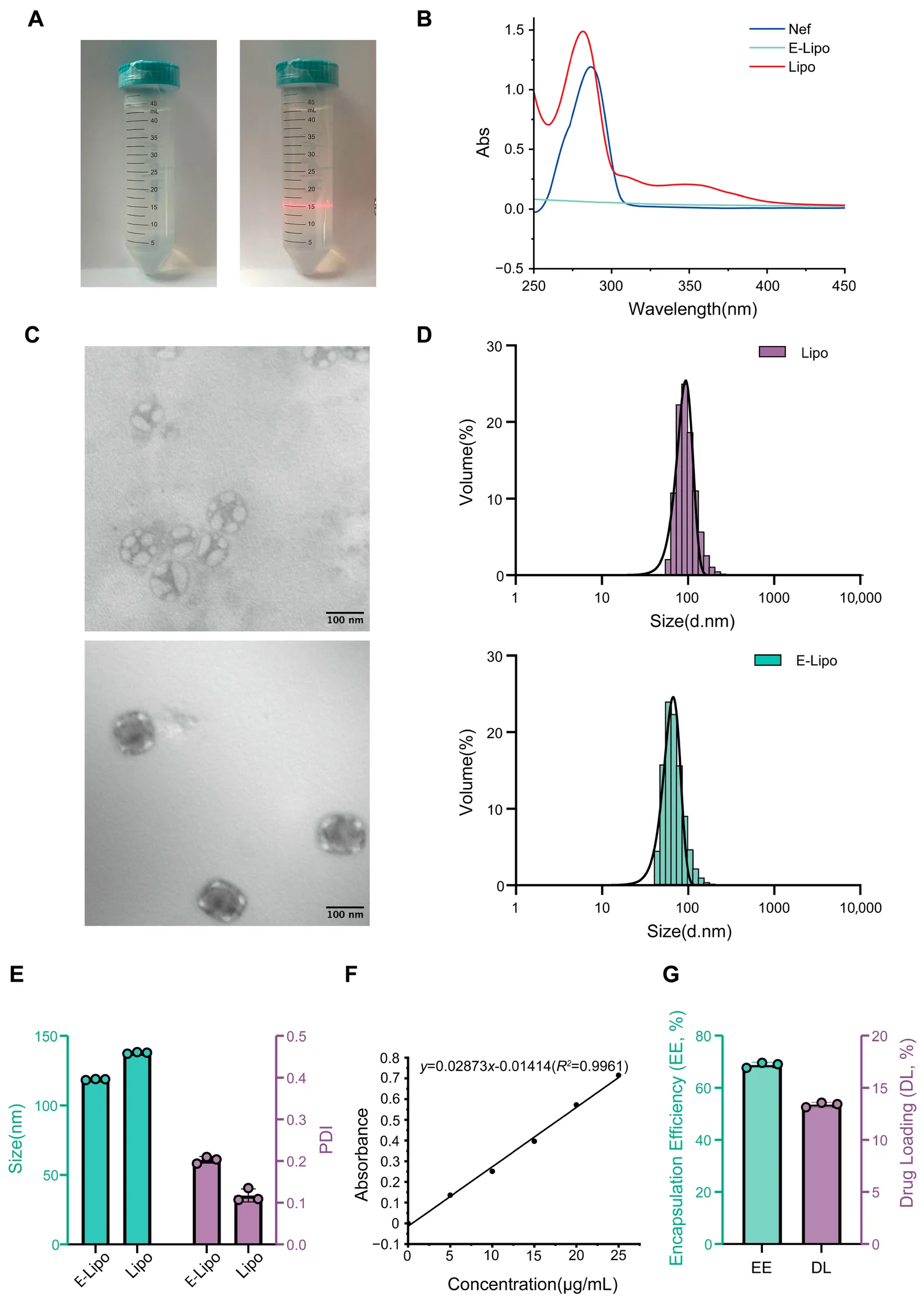
Characterization of Lipo. (**A**) Appearance of Lipo and demonstration of the Tyndall effect. (**B**) UV absorption spectra of free Nef, empty liposomes (E-Lipo), and Lipo. (**C**) Transmission electron microscopy (TEM) images of E-Lipo and Lipo; (scale bar = 100 nm). (**D**) Particle size distribution of Lipo and E-Lipo. (**E**) Average particle size and polydispersity index (PDI) of Lipo and E-Lipo. (**F**) Standard calibration curve of Nef. (**G**) Encapsulation efficiency (EE) and drug loading (DL) of Lipo (*n* = 3).

Lipo prepared by the TFH method exhibited a clear, opalescent appearance with a visible Tyndall effect, confirming their nanoscale characteristics (Figure 2A). As shown in Figure 2B, free Nef displayed a characteristic absorption peak at 287 nm, whereas Lipo exhibited an absorption maximum at approximately 280 nm after methanol treatment. No corresponding peak was observed for empty liposomes (E-Lipo), confirming the presence of Nef in the liposomes.

Transmission electron microscopy (TEM) images revealed that both Lipo and E-Lipo were spherical or nearly spherical with good dispersion (Figure 2C). E-Lipo exhibited a hollow structure with low internal electron density, whereas Lipo showed higher internal density, consistent with drug loading. The dynamic light scattering (DLS) analysis (Figure 2D,E) showed that the mean hydrodynamic diameters of Lipo and E-Lipo were 137.90 ± 0.56 nm and 118.80 ± 0.42 nm, respectively. The PDI values for Lipo and E-Lipo were 0.117 ± 0.016 and 0.203 ± 0.008, respectively, indicating a narrow size distribution.

The Nef calibration curve showed excellent linearity (*R*^2^ ≥ 0.99) for drug quantification (Figure 2F). Based on this calibration curve, the EE and drug loading (DL) of Lipo were determined to be 68.79 ± 1.01% and 13.38 ± 0.22%, respectively (Figure 2G), indicating efficient encapsulation of the hydrophobic alkaloid. Pre-saturation of the ultrafiltration membrane with E-Lipo significantly reduced non-specific drug adsorption, ensuring the accuracy of the EE measurements.

### 2.2. Ex Vivo Transdermal Permeation

**Figure 3:**
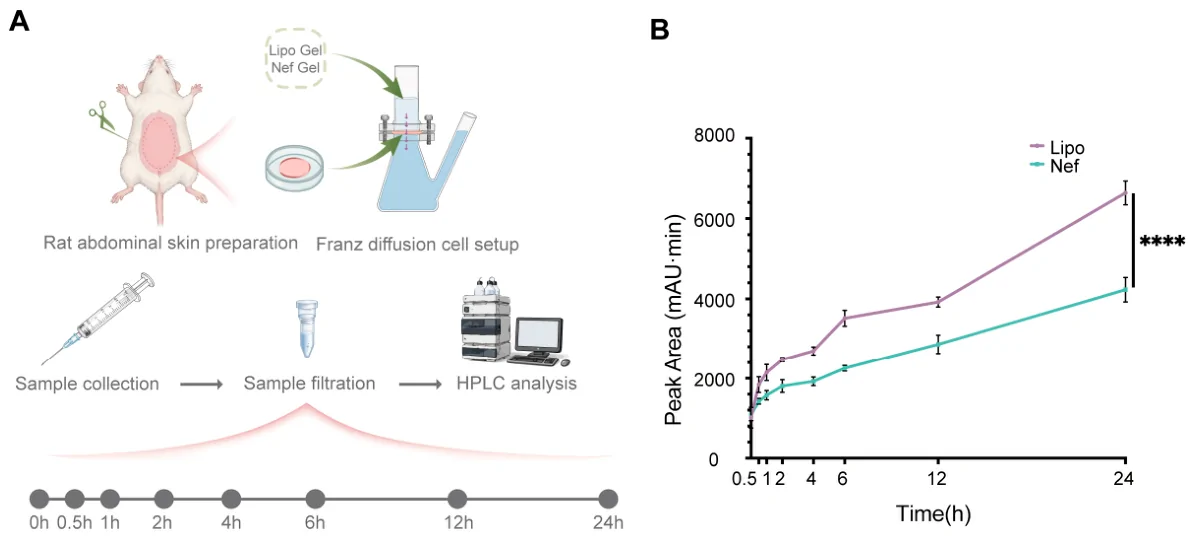
Ex vivo skin permeation. (**A**) Schematic of the ex vivo skin permeation protocol. (**B**) Comparison of cumulative transdermal permeation profiles of Nef and Lipo over 24 h (*n* = 3). Statistical significance: **** *p* < 0.0001.

A high-performance liquid chromatography (HPLC) method with a standard curve of *y* = 6359.4*x* + 4147.2 (*R*^2^ = 0.9941) was established for Nef quantification. The Franz diffusion cell assay (Figure 3A,B) revealed that both free Nef and Lipo exhibited rapid penetration during the initial 0.5–2 h. Lipo showed a greater overall increase in cumulative permeation than free Nef. Significant differences between the two groups were observed from 2 h onwards (*p* < 0.05), and the cumulative permeation of Lipo after 24 h was significantly higher than that of free Nef (*p* < 0.0001). This demonstrates that liposomal encapsulation significantly enhances and prolongs the transdermal delivery of Nef.

### 2.3. In Vivo Anti-Photoaging Efficacy

**Figure 4:**
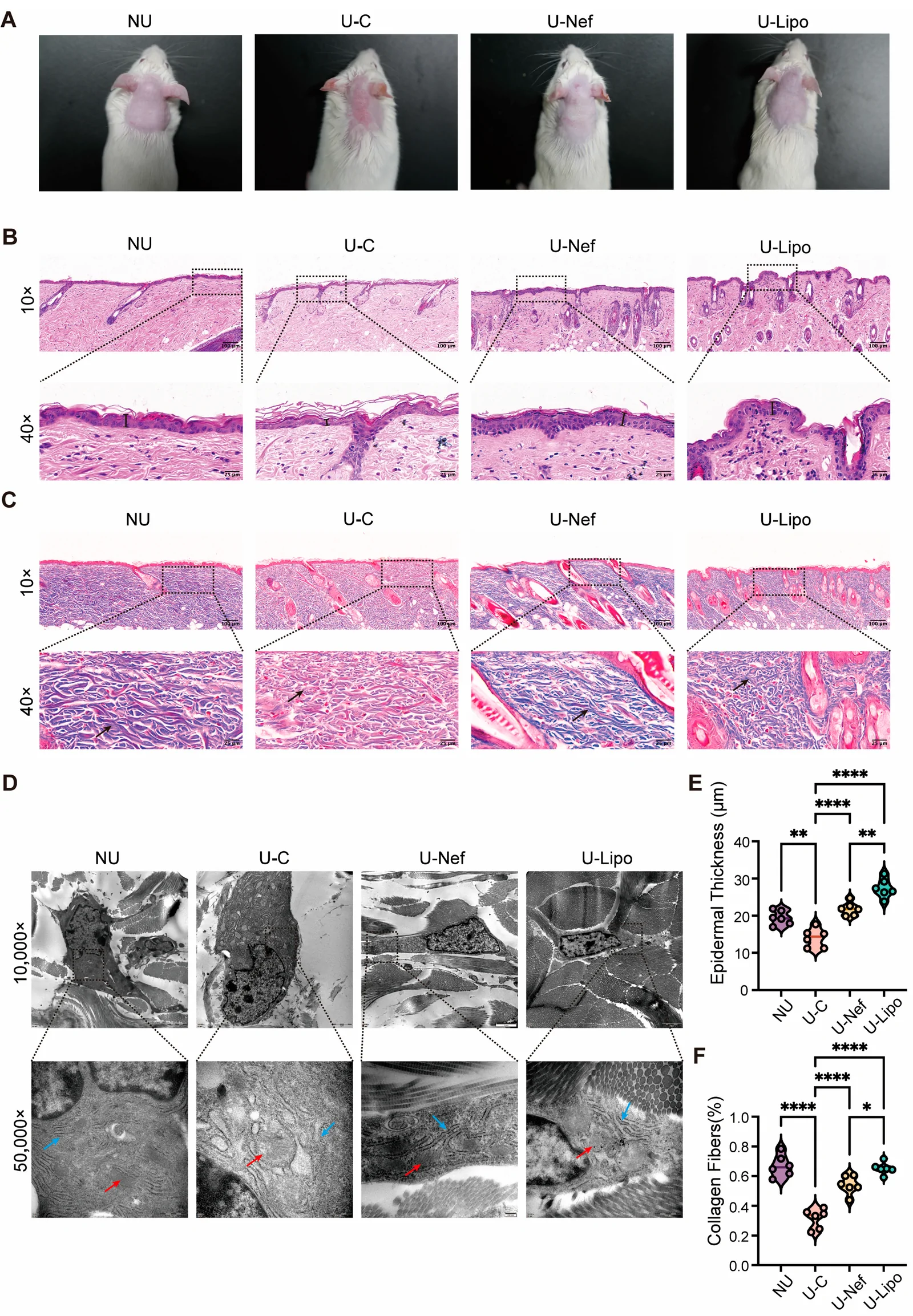
Histological analysis of the anti-photoaging effects of Lipo. (**A**) Representative photographs of dorsal skin for 6 weeks. (**B**) Hematoxylin and eosin (H&E) staining (scale bars: 100 μm (upper) and 25 μm (lower)); short bars indicate the epidermis. (**C**) Masson’s trichrome staining (scale bars: 100 μm (upper) and 25 μm (lower)); arrows indicate collagen fibers. (**D**) TEM images of dermal fibroblasts (magnifications: 10,000× and 50,000×); blue arrows indicate the endoplasmic reticulum, and red arrows indicate mitochondria. (**E**) Quantitative analysis of epidermal thickness. (**F**) Quantitative analysis of collagen fiber area. Data are mean ± standard deviation (SD) (*n* = 6). Statistical significance: * *p* < 0.05, ** *p* < 0.01, **** *p* < 0.0001.

As shown in Figure 4A, the UVA + blank gel (U-C) group exhibited pronounced photoaging features, including erythema, roughness, and wrinkle formation, confirming the successful establishment of the chronic photoaging model. In contrast, both the UVA + 0.1% Nef gel (U-Nef) and UVA + 0.1% Lipo gel (U-Lipo) groups showed significantly less skin damage, with the U-Lipo group displaying superior protection.

Histological analysis via hematoxylin and eosin (H&E) (Figure 4B,E) revealed that UVA irradiation caused significant epidermal thinning and loss of stratified squamous epithelium in the U-C group. Treatment with Nef or Lipo effectively reversed these changes, significantly restoring epidermal thickness. Masson’s trichrome staining (Figure 4C,F) demonstrated a marked reduction and disorganization of collagen fibers in the U-C group. In contrast, the U-Nef and U-Lipo groups showed increased collagen content and better-organized fibrillar networks. Notably, the collagen content in the U-Lipo group was slightly higher than in the U-Nef group, suggesting enhanced efficacy via liposomal delivery.

TEM analysis of dermal fibroblasts (Figure 4D) showed that UVA exposure induced severe ultrastructural damage, characterized by swollen, vacuolated mitochondria with disrupted cristae, and dilated, fragmented endoplasmic reticulum (ER). In the drug-treated groups, particularly in U-Lipo, mitochondrial morphology was substantially preserved, cristae were clearly visible, and ER structure was restored. These results confirm that liposomal encapsulation of Nef provides superior protection against UVA-induced photoaging at the tissue and cellular level.

### 2.4. Lipo Inhibits Cellular Senescence and Oxidative Stress In Vitro

#### 2.4.1. Cell Viability and Senescence

**Figure 5:**
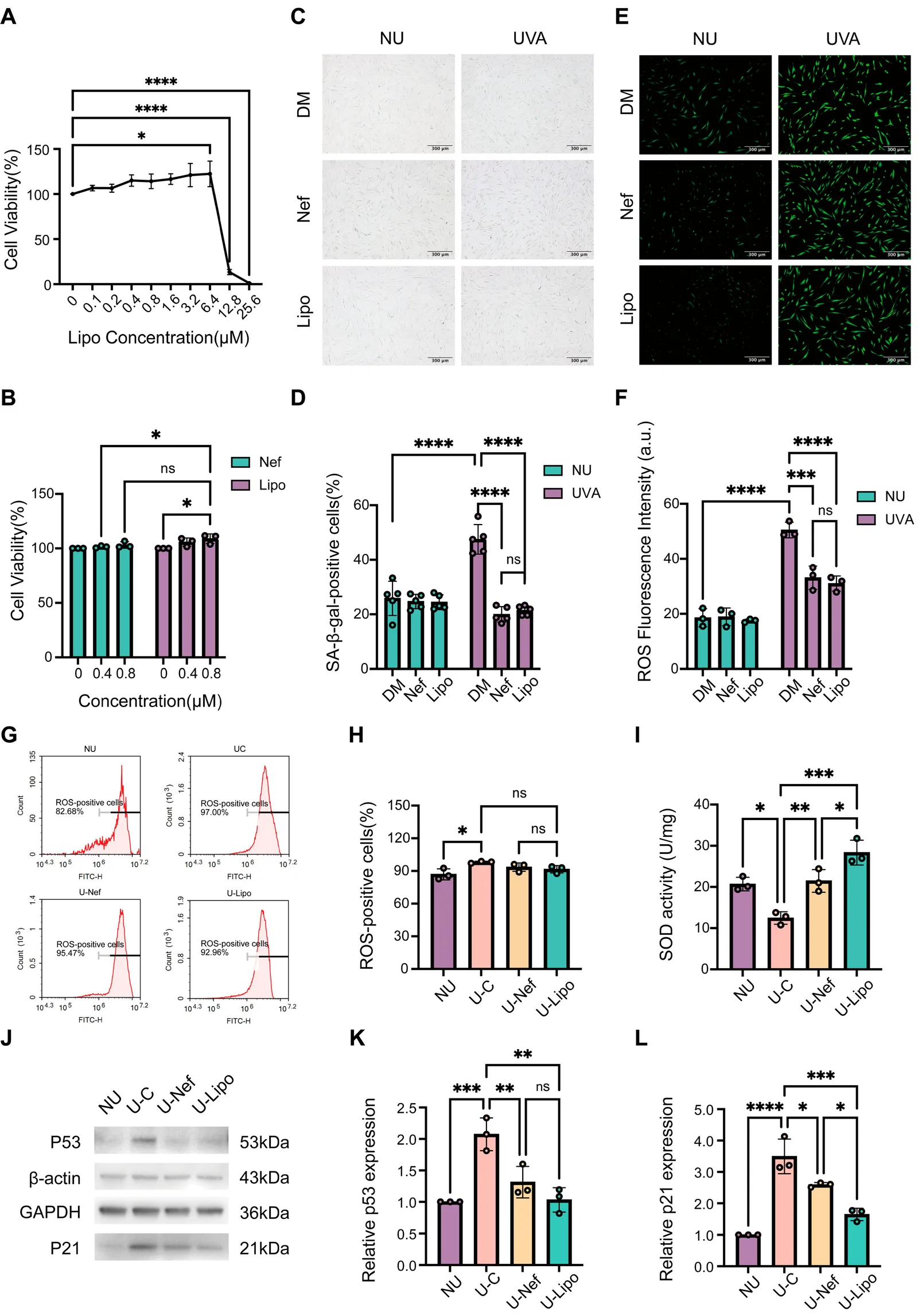
In vitro validation of Lipo. (**A**) Cell viability of fibroblasts after 24 h treatment with a gradient of liposomal concentrations. (**B**) Comparison of cell viability between Nef and Lipo at 0.4 μM and 0.8 μM. (**C**) Representative images of Senescence-Associated β-Galactosidase (SA-β-gal) staining (scale bar = 300 μm). (**D**) Quantification of SA-β-gal-positive cells. (**E**) Representative fluorescence images of intracellular ROS levels (scale bar = 300 μm). (**F**) Quantitative analysis of ROS fluorescence intensity. (**G**) Representative flow cytometry histograms of intracellular ROS. (**H**) Quantification of ROS-positive cell percentage. (**I**) SOD activity in each group. (**J**) Representative Western blot bands showing the protein expression levels of p53 and p21. (**K**,**L**) Quantitative analysis of p53 and p21 protein expression normalized to internal control. Data are mean ± SD. Statistical significance: ns, not significant; * *p* < 0.05, ** *p* < 0.01, *** *p* < 0.001, **** *p* < 0.0001.

As shown in Figure 5A, Lipo at concentrations of 0.1–6.4 μM promoted fibroblast proliferation in a dose-dependent manner. However, at concentrations exceeding 6.4 μM, cytotoxicity was observed, with cell viability significantly reduced at 12.8 μM and 25.6 μM (*p* < 0.0001). At equivalent concentrations (0.4 and 0.8 μM), Lipo tended to exhibit higher cytocompatibility and enhanced bioactivity compared to free Nef (Figure 5B).

The Senescence-Associated β-Galactosidase (SA-β-Gal) staining (Figure 5C,D) revealed a significant increase in senescent cells in the UVA-irradiated model group cultured in complete DMEM without drug treatment (UVA-DM) group compared to the blank control (NU) group (*p* < 0.0001). Treatment with UVA-Nef or UVA-Lipo significantly reduced the proportion of SA-β-Gal-positive cells, indicating that Nef effectively counteracts UVA-induced cellular senescence, with liposomal encapsulation producing a comparable effect to free Nef.

#### 2.4.2. Antioxidant Effects

UVA exposure significantly increased intracellular ROS levels, as indicated by elevated DCFH-DA fluorescence (Figure 5E–H). Treatment with Nef and Lipo significantly attenuated ROS accumulation. Concomitantly, UVA irradiation caused a significant decline in SOD activity, which was effectively restored upon drug treatment (Figure 5I). The U-Lipo group showed a more pronounced recovery of SOD activity than the U-Nef group (*p* < 0.05). These results confirm that Nef alleviates UVA-induced oxidative stress by scavenging ROS and restoring endogenous antioxidant enzyme activity, with liposomal delivery system showing a modest advantage in restoring SOD activity.

### 2.5. Lipo Regulates the p53/p21 Pathway to Inhibit Cellular Senescence

To elucidate the underlying molecular mechanism, the expression of p53 and p21 was examined by Western blotting (Figure 5J–L). UVA irradiation significantly upregulated p53 and p21 expression in the model group compared to NU. However, treatment with Nef and Lipo markedly suppressed this upregulation. These findings indicate that Nef inhibits UVA-induced cellular senescence by downregulating the p53/p21 signaling axis, with liposomal encapsulation showing a modest additional effect on p21 expression.

## 3. Discussion

In this study, we successfully developed a Nef-loaded liposomal delivery system using the TFH method and systematically evaluated its physicochemical properties, transdermal behavior, and protective efficacy against skin photoaging. The results demonstrated that Lipo not only possessed favorable pharmaceutical characteristics, including nanoscale particle size, narrow size distribution, and high EE, but also significantly enhanced the transdermal permeation of Nef and exhibited superior photoprotective activity compared to free Nef in both in vivo and in vitro models. These findings suggest that liposomal nanoencapsulation addresses the bioavailability bottleneck associated with transdermal delivery of Nef and preserves its beneficial effects on oxidative stress and cellular senescence pathways, offering a formulation strategy with translational potential.

Liposomes are spherical vesicles formed by the self-assembly of phospholipid bilayers, capable of encapsulating both hydrophilic and hydrophobic therapeutics [28]. Owing to their structural versatility, excellent biocompatibility, and controllable drug release profiles, liposomes are one of the most extensively studied nanocarrier platforms [29]. Their properties are primarily governed by lipid composition and fabrication methodology. The TFH method is the most widely adopted due to its operational simplicity, cost-effectiveness, and compatibility with diverse lipid systems [30]. In our formulation, DSPC provides membrane rigidity due to its high phase transition temperature (Tm ≈ 55 °C), which reduces drug leakage [31]. Cholesterol intercalates between acyl chains, reducing membrane permeability and enhancing structural integrity [32]. DSPE-PEG2000 forms a hydrophilic steric barrier that can improve liposomal colloidal stability and may enhance stratum corneum hydration, potentially contributing to skin permeation [33,34]. The selected lipid composition (DSPC: cholesterol ≈ 2:1) has been shown to optimally balance bilayer integrity with drug encapsulation capacity [32].

Physicochemical characterization confirmed that Lipo possessed a unilamellar vesicular morphology with narrow size distribution (PDI ≤ 0.3) [35]. The mean hydrodynamic diameter of ~140 nm is within the optimal size window for skin penetration via the follicular route, allowing the formation of a localized drug depot for sustained release [36,37,38,39]. The modest size increase upon drug loading, together with the high EE (68.79%) and DL (13.38%), supports the successful loading of Nef into the liposomes and indicates strong physicochemical compatibility between the rigid DSPC/cholesterol bilayer and the hydrophobic alkaloid [40,41]. Future studies should, however, determine the precise localization of Nef within the bilayer and assess long-term colloidal stability under storage conditions to support clinical translation.

The ex vivo Franz diffusion cell experiments revealed phase-dependent transdermal permeation kinetics [42]. During the initial phase (0.5–2 h), both free Nef and Lipo exhibited rapid permeation. Thereafter, the permeation rate of both formulations decreased, but Lipo showed greater cumulative permeation than free Nef, resulting in significantly higher cumulative permeation at 24 h. This sustained release profile may be attributed to the follicular drug reservoir mechanism and the occlusive effect of the liposomal components, which reduces stratum corneum barrier resistance [39]. This kinetic profile provides a direct mechanistic basis for the superior in vivo efficacy of Lipo. We acknowledge that the use of ex vivo skin presents inherent limitations and that future studies with advanced human skin models (e.g., 3D bioprinted constructs) are warranted.

Both in vivo and in vitro experiments confirmed that the anti-photoaging effects of Nef are mediated by blocking the “ROS/SOD” oxidative stress cascade and the downstream “p53/p21” senescence pathway. UVA-induced mitochondrial ROS overload leads to endogenous SOD depletion and DNA damage, which stabilizes and activates the p53/p21 cascade, ultimately driving cell cycle arrest and the senescent phenotype [43,44]. Nef effectively interrupted this cascade. The Lipo group demonstrated superior outcomes, including higher SOD activity, greater downregulation of p21, and enhanced histological repair, directly attributable to the higher effective drug concentration achieved through the liposomal delivery system. These findings align with previous studies showing that nanoliposomes can enhance the therapeutic efficacy of bioactive compounds by improving drug stability, facilitating skin penetration, and prolonging local release. Nevertheless, as the empty liposome (E-Lipo) was not tested under UVA exposure, the observed effects are best interpreted as arising from the Nef-loaded liposomal formulation as a whole, and the individual contribution of the carrier remains to be clarified.

## 4. Materials and Methods

### 4.1. Materials

Nef (purity ≥ 98%) was purchased from Pure One Biotechnology Co., Ltd. (Shanghai, China). DSPC, DSPE-PEG2000, and DSPE-PEG2000-COOH were obtained from Pengsheng Biotechnology Co., Ltd. (Shanghai, China). Cholesterol was purchased from Aladdin Biochemical Technology Co., Ltd. (Shanghai, China). All other chemicals were of analytical grade.

Female Kunming (KM) mice (*n* = 40, 8–10 weeks old, 25 ± 2 g) and male Sprague-Dawley (SD) rats (*n* = 3, 6–8 weeks old, 160 ± 20 g) were obtained from the Experimental Animal Center of Xi’an Jiaotong University (Xi’an, China). All animals were housed under standard conditions (12-h light/dark cycle, free access to food and water) for 1 week prior to experiments. All procedures were approved by the Biomedical Ethics Committee of Health Science Center of Xi’an Jiaotong University.

### 4.2. Preparation of Lipo

Lipo were prepared using the TFH method. Briefly, Nef (200 mg in 10 mL methanol) was mixed with lipids (DSPC, cholesterol, DSPE-PEG2000, and DSPE-PEG2000-COOH at a 2:1:0.11:0.021 molar ratio) dissolved in 40 mL chloroform. The organic solvents were removed under reduced pressure using a rotary evaporator (RV 10 digital, IKA, Staufen, Germany) to form a thin lipid film on the wall of a round-bottom flask. The dried film was hydrated with phosphate-buffered saline (PBS, pH 7.4) under stirring for 1 h, followed by sonication for 20 min to reduce vesicle size. The resulting dispersion was extruded through a 0.45 μm polycarbonate membrane. E-Lipo were prepared identically but without the addition of Nef [45].

### 4.3. Physicochemical Characterization of Liposomes

#### 4.3.1. UV-Vis Spectrophotometry

The UV absorption spectra of free Nef, Lipo, and E-Lipo were recorded using a TU-1810 spectrophotometer (Beijing Purkinje General Instrument, Beijing, China) over a wavelength range of 250–450 nm. Samples were diluted in methanol, using methanol as a blank.

#### 4.3.2. Morphological Analysis

The morphology of Lipo and E-Lipo was visualized by TEM (JEM-200CX, JEOL, Akishima, Japan). A drop of each sample (10 μL) was placed onto a carbon-coated copper grid, incubated for 30 s, and the excess solution was removed. The grids were air-dried overnight before imaging.

#### 4.3.3. Particle Size and PDI

The hydrodynamic diameter and PDI of Lipo and E-Lipo were measured using DLS on a Zetasizer Nano ZS90 (Malvern Instruments, Malvern, UK). Each sample was measured in triplicate at 25 °C.

#### 4.3.4. EE and DL

The drug encapsulation and loading capacity were determined using an ultrafiltration-centrifugation method. Briefly, to minimize non-specific adsorption, the ultrafiltration tubes (30 kDa MWCO) were pre-saturated with E-Lipo. A 0.5 mL aliquot of Lipo was then placed into the pre-treated tube and centrifuged at 3000× *g* for 3 min at 4 °C. The concentration of free Nef in the filtrate (*W_free_*) was quantified by UV-Vis spectrophotometry at 287 nm. The total Nef content (*W_total_*) was measured by dissolving 0.1 mL of Lipo in methanol, followed by ultrasonication. The EE and DL were calculated using the following equations:

(1) $$EE(\%)=\frac{W_{\mathrm{total}}-W_{\mathrm{free}}}{W_{\mathrm{total}}}\times 100\%$$

(2) $$\mathrm{DL}\left(\%\right)=\frac{W_{\mathrm{entrapped}}}{W_{\mathrm{lipo}}-W_{\mathrm{entrapped}}}\times 100\%$$

where *W_entrapped_* = *W_total_* − *W_free_*, and *W_lipo_* is the total weight of the lyophilized liposome powder. All experiments were conducted in triplicate, and the results are presented as mean ± SD.

### 4.4. In Vivo Animal Experiments

#### 4.4.1. UVA Irradiation in Animal Experiments

A 1.5% (*w*/*v*) carbomer gel was prepared, pH-adjusted, and stored at 4 °C. Nef or Lipo was incorporated into the gel to achieve a final concentration of 0.1% (*w*/*v*). Twenty-four female KM mice were depilated on the dorsal area and randomly divided into four groups (*n* = 6 per group): (1) NU (no UVA and no treatment); (2) U-C; (3) U-Nef; and (4) U-Lipo. UVA exposure (320–400 nm) was performed five times per week for six weeks, with a total irradiation dose of 1800 min. Photoaging was assessed by gross visual observation of the dorsal skin. The model endpoint was defined as the point at which visible changes in skin appearance, including redness, surface coarseness, and wrinkling, were evident in the U-C group relative to the control group. At the end of the experiment, mice were euthanized, and dorsal skin samples were harvested for further analysis.

#### 4.4.2. Histological Analysis

Skin samples were fixed in 4% paraformaldehyde, embedded in paraffin, and sectioned at 4 μm thickness. For histological evaluation, sections were stained with H&E to assess epidermal thickness and with Masson’s trichrome to visualize collagen fibers. Images were captured under a light microscope, and epidermal thickness and collagen area were quantified using ImageJ software 1.54g (NIH, Bethesda, MD, USA).

#### 4.4.3. TEM of Skin

To observe fibroblast ultrastructure, small pieces of skin from each group were fixed in glutaraldehyde and processed for TEM analysis. Ultrathin sections were examined under a transmission electron microscope.

### 4.5. In Vitro Experiments

#### 4.5.1. Cell Culture and UVA Irradiation Model

Primary human dermal fibroblasts were isolated from eyelid skin of healthy female volunteers (20–40 years old) and cultured in DMEM supplemented with 10% FBS and 1% penicillin-streptomycin at 37 °C in a 5% CO_2_ atmosphere. Cells at passages 3–8 were used for all experiments. To establish an in vitro photoaging model, fibroblasts were exposed to UVA irradiation (≈10 J/cm^2^/day) for 7 consecutive days in DMEM containing 1% FBS, with or without Nef or Lipo treatment [46].

#### 4.5.2. Cell Viability Assay

Cell viability was assessed using the CCK-8 assay. Fibroblasts were seeded in 96-well plates (6000 cells/well) and treated with various concentrations of Lipo (0.1–12.8 μM) or Nef (0.4 and 0.8 μM) for 24 h. Following treatment, CCK-8 solution was added to each well, incubated for 4 h, and the absorbance was measured at 450 nm.

#### 4.5.3. SA-β-Gal Staining

Based on initial screening, 0.8 μM Lipo was selected for subsequent experiments. After UVA exposure, cells were washed, fixed, and incubated with SA-β-Gal staining solution (Beyotime, Shanghai, China) at 37 °C (without CO_2_) for 24 h. Stained cells were imaged under a light microscope, and the percentage of SA-β-Gal-positive cells was quantified.

#### 4.5.4. Measurement of Intracellular ROS

Intracellular ROS levels were measured using the DCFH-DA probe. After UVA exposure, cells were incubated with 10 μM DCFH-DA at 37 °C for 20 min, washed, and analyzed by fluorescence microscopy and flow cytometry (FITC channel, Agilent, Santa Clara, CA, USA).

#### 4.5.5. Measurement of SOD Activity

Fibroblasts from all experimental groups were lysed, and SOD activity in the supernatant was determined using a Total SOD Assay Kit (WST-8 method, Beyotime, Shanghai, China). Total protein concentration was measured using a BCA assay kit (NCM, Suzhou, China). SOD activity was expressed as U/mg protein.

#### 4.5.6. Western Blotting

Total protein was extracted from cells using RIPA buffer containing 1% PMSF. Equal amounts of protein were separated by SDS-PAGE and transferred to PVDF membranes. Membranes were blocked and incubated overnight at 4 °C with primary antibodies against p53, p21, GAPDH, and β-actin (ABclonal, Woburn, MA, USA). After incubation with HRP-conjugated secondary antibodies, protein bands were visualized by chemiluminescence and quantified using ImageJ.

### 4.6. Ex Vivo Transdermal Permeation Study

An HPLC method was developed and validated for the quantification of Nef. The chromatographic conditions were: C18 column (150 × 4.6 mm, 5 μm); detection wavelength, 282 nm; mobile phase, acetonitrile and 0.01 M sodium acetate (containing 0.4 g sodium dodecyl sulfate per 100 mL, pH adjusted to 3.0 with phosphoric acid) (52:48, *v*/*v*); flow rate, 1 mL/min; column temperature, 40 °C; injection volume, 10 μL.

Full-thickness skin from the abdominal region of SD rats was excised, cleaned of subcutaneous tissue, and mounted on Franz diffusion cells with the dermal side facing the receptor chamber. The donor chamber was filled with 2 mL of either Nef or Lipo formulation (0.1% *w*/*v* in carbomer gel). The receptor chamber was filled with PBS (pH 7.4) containing 5% (*v*/*v*) ethanol to maintain sink conditions. The system was maintained at 37 °C with constant stirring. At predetermined time points (0.5, 1, 2, 4, 6, 12, and 24 h), 1 mL of receptor medium was withdrawn and replaced with an equal volume of fresh pre-warmed medium. The concentration of Nef in the samples was determined by HPLC.

### 4.7. Statistical Analysis

All data are presented as mean ± SD. Statistical analyses were performed using GraphPad Prism 10.3.1 (GraphPad Software, San Diego, CA, USA). Comparisons between multiple groups were performed using one-way or two-way analysis of variance (ANOVA), followed by appropriate post hoc tests. A *p*-value < 0.05 was considered statistically significant.

## 5. Conclusions

This study substantiates the therapeutic value of Nef in attenuating skin photoaging and validates the advantage of liposomal encapsulation in enhancing the transdermal efficacy of this bioactive natural compound. Compared to the free drug, the Lipo system optimized drug stability, transdermal permeability, and local tissue retention, effectively overcoming the bioavailability bottleneck associated with hydrophobic natural products. Future investigations should incorporate biomimetic skin models, conduct systematic long-term stability assessments, and compare carboxyl-bearing liposomes with conventional PEGylated liposomes to clarify the contribution of the carboxyl groups and explore their potential for surface functionalization, so as to advance the clinical translation and scalable manufacturing of this nanoplatform.

## Data Availability

The original contributions presented in this study are included in the article. Further inquiries can be directed to the corresponding authors.
